# Immune gene expression in salmon keratocytes upon bacterial exposure

**DOI:** 10.1186/s12860-025-00553-9

**Published:** 2025-09-24

**Authors:** Marie Kristin Mikkelborg, Antonette Skram Helgestad, Roy Ambli Dalmo, Dhivya Borra Thiyagarajan

**Affiliations:** Norwegian College of Fishery Science, Faculty of Biosciences, Fisheries and Economics, UiT the Arctic University of Norway, Tromsø, Norway

**Keywords:** Atlantic salmon, Salmon skin keratocytes (SKCs), Corneal keratocytes (CKCs), Pro-inflammatory cytokines, Phagocytic markers, Fish pathogens

## Abstract

**Background:**

Pathogens pose a significant threat to farmed salmon, adversely affecting their health and welfare, which results in substantial economic losses. The outermost skin epithelial cells, including skin keratocytes, serve as a critical defense barrier against environmental stressors, such as bacterial infections. Understanding the features and properties of these cells, particularly skin keratocytes, is essential as they play a crucial role in wound healing and tissue regeneration. Additionally, we were curious whether corneal epithelial cells (corneal keratocytes), which protect the fish eye, exhibit similar features and immune functions.

**Results:**

To investigate the properties of skin keratocytes, we utilized flow cytometry to analyze scale-derived cells. The analysis revealed that more than 85% of the cells were skin keratocytes, although the remaining cells could not be characterized using this method. Microscopic analysis confirmed that the majority of these cells exhibited the typical morphology of keratocytes and were positive for a pan-keratin antibody. To assess the immune function of skin keratocytes, we exposed scale-derived keratocytes to various fish pathogenic bacteria and evaluated their ability to ingest bacteria and examined the regulation of innate immune genes. Additionally, we analyzed the expression of immune-related markers, including *mhcii*, in both skin and corneal cells.

**Conclusion:**

Our findings demonstrated that scale-derived skin keratocytes internalized bacteria within 3–6 hours of exposure. This bacterial interaction led to the significant upregulation of several key immune-related genes. Interestingly, both skin and corneal cells expressed *mhcii,* suggesting a potential role in adaptive immunity in addition to their participation in innate immune responses. These results highlight the vital role of skin and corneal cells in the innate immune response against bacterial infections and their possible contribution to adaptive immunity.

**Supplementary information:**

The online version contains supplementary material available at 10.1186/s12860-025-00553-9.

## Introduction

The innate immune system in vertebrates is non-specific, meaning it generally responds to a wide range of pathogens without recognizing specific pathogens or antigens [1]. The innate immune response in *Salmo salar* (and other fish species) forms the first line of defense against pathogens [2]. Salmon skin and their cells make up an important innate defense barrier against pathogens [3]. In particular, skin epithelial cells show high motility speed and are involved in phagocytosis as well as rapid wound healing, which is vital for the fish´ survival in a pathogen-rich environment [4–7]. The skin epithelium consists mainly of non-leucocytic cells whereas the sub-dermis contains various immune cells, such as macrophages and neutrophils [8]. The skin cells are known to respond to pathogens such as bacteria, viruses and parasites [3, 9–11], even though it is not known which cells are the central ones in these responses. The corneal layer of the eye is also known to protect the eye from e.g., pathogens [4]. As in skin, the outermost layer of cornea of fish also contains epithelial cells (corneal keratocytes) which are highly similar, morphologically, to skin keratocytes [12]. The corneal keratocytes are also phagocytic [12, 13], ingesting e.g., plastic microparticles. Besides keratocytes, it has been shown that leucocytes are present in the fish corneal layer suggesting a potency for an immune response [14]. The exact location of these leucocytes was not addressed. Corneal keratocytes have been shown to respond to virus infection [14], bacteria [15], and parasites [16]. In summary, it is not known how the outermost epithelial cells in skin and eye respond to bacteria with respect to phagocytosis or immune response. It is known that so-called non-professional phagocytes are present in fish, where B-cells and thrombocytes have been found to phagocytose e.g., bacteria or particles [17, 18]. Non-professional phagocytes have limited phagocytic capacity but may interact with leucocytes to induce immune response [19]. In higher vertebrates, it is also clear that epithelial cells are sentinels with phagocytic properties and can induce immune responses [20]. As the skin and corneal cells can phagocytose bacteria and particles it is likely that these cells also are non-professional phagocytes.

Following this, we hypothesize that skin and corneal keratocytes are non-professional phagocytes which respond to bacterial exposure by regulation of immune-related genes. Our findings revealed that salmon keratocytes can ingest bacteria within 3–6 hours of exposure following regulation of immune-related genes. This offers a foundation for further investigation into the cellular mechanisms, such as immune receptors, that underlie immune responses to pathogens. Understanding how host cells interact with pathogens could open new avenues for exploring cell-to-cell communication strategies in keratocytes.

## Materials and methods

### Ethical statement

Atlantic salmon (*Salmo salar* L.) used in this study were post-smolts (*ca*. 0.5–2 kg) and obtained from the Tromsø Aquaculture Station, Tromsø, Norway. In this research facility, the fish were fed commercial diets in flow through concrete 5000 L. The fish were clinically healthy and free from pathogens – as routine diagnostics were carried out regularly. Fish were euthanized by a sharp blow to the head, and the fish were immediately transported on ice to UiT - the Arctic University of Norway. This handling of fish, and picking of scales do not require special approval, as detailed in the Norwegian Regulations for use of animals in experimentation (https://lovdata.no/dokument/SF/forskrift/2015-06-18-761#KAPITTEL_10). This method is also in compliance with corresponding EU legislation—Directive2010/63/EU(https://eur-lex.europa.eu/legal-content/EN/TXT/?uri=CELEX:32010L0063).

### Antibodies/chemical

The cells were incubated in “HBBS mix” where 10 ml of 1000 U penicillin/100 µg streptomycin mix and 100 µg of amphotericin (HBSS mix) were added per liter of HBSS. Rabbit Pan keratin antibody (KRT/1877 R), Alexa 488 anti-rabbit IgG (A-11008), propidium iodide (eBioscience- 00–6990–50), Lysotracker Deep Red (LTDR-L12492) and DAPI (11530306) were purchased from ThermoFisher Scientific (USA). BactoView live fluorescent bacteria stains from Biotium (40102-T) was used to stain live bacteria for visualization of bacterial internalization in keratocytes.

### Cell isolation

Keratocytes from both scales and cornea were obtained in the lab like previous studies [12]. Briefly, for skin keratocytes, individual scales were pulled from skin using clean forceps and placed in either cell culture dishes or glass bottom dishes according to the requirement. The scales were left in the dish for 6–10 minutes before adding HBSS mix. About 2–3 days after scale harvesting and seeding (4^◦^C) cell sheets were formed from cell avalanches. These cell sheets and single cells were used for microscopic analysis and RNA extraction. Corneal epithelial cell isolation: The individual cornea was carefully removed and cleaned using HBSS mix before the corneas were placed upside down in the culture dish. After 15 minutes, HBSS mix was added, and the corneas were incubated at 4^◦^C until the cells were migrating from cornea – as cell sheets or individual cells. Cell sheets and individual cells were analyzed as described below.

### Bacterial culture and infection

**Fig. 1:**
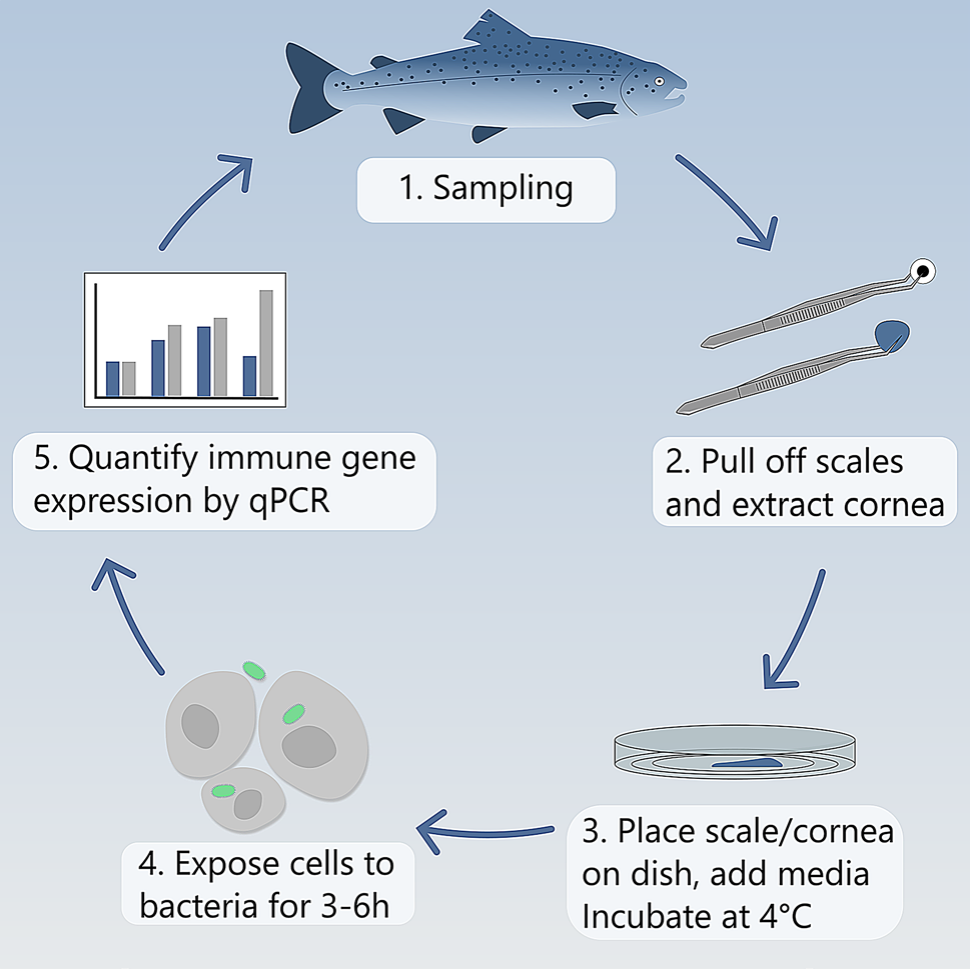
Graphical illustration of cell isolation and bacterial exposure

Four different fish pathogen strains were used in this study. Glycerol stocks of *Moritella viscosa*, *Aeromonas salmonicida*, *Vibrio anguillarum*, and *Vibrio salmonicida* were obtained from Nofima as a kind gift. These have been described before [21]. Glycerol stocks were streaked onto blood agar plates with 2%NaCl (provided by National Veterinary Institute, Ås, Norway) and incubated at 12^◦^C until colony formation. At approximately 48 hours colonies were formed from all the strains; these colonies were tested to confirm the presence of respective bacterial species using the Mono aqua-test system (Bionor laboratories AS). After confirmation of each strain, single colonies were picked and grown in liquid media. *M. viscosa* and *V. salmonicida* grew well in FAMP media (5 g tryptone,15 g Marine broth,700 ml Milli-Q water, 300 ml saltwater) while *V. anguillarum* and *A. salmonicida* grew well in MH media (21 g Mueller Hinton Broth (Difco) in 1 L Milli-Q water). Once OD reached OD ~ 0.6–0.8, bacterial cells were harvested by centrifuging at 10,000 rpm at 4^◦^C for 10 minutes. Cell pellets were washed using 0.9% NaCl before the bacteria were suspended in HBSS. After washing, the bacterial pellets were re-suspended and diluted to 10^−2^/ml and this dilution which was used in further experiments. Figure 1 shows a graphical design illustrates the detailed process of cell isolation and bacterial exposure.

### Flow cytometry

Skin keratocyte were grown in culture dishes until they reached 70% confluence, then cells were de-attached from culture dishes using accutase (BioWset – L0950). After accutase treatment, the cells were centrifuged at 800 *g* for 10 minutes. The cell pellet was suspended in HBSS mix, and the cells were counted using a Nucleocounter slide (941–0001). Keratocytes were stained with Pan keratin antibody for 1 hour at RT, washed and stained with Alexa 488 antibody. The pellets were resuspended in HBSS to give 5.0 × 10^4^ cells/mL for the flow cytometry analysis using the BD Fortessa flow cytometer (BD Biosciences). Dead cells (PI^+^) were excluded from the population and analysis. The remaining cells were gated based on their forward scatter (FSC) and side scatter (SSC) profiles, and with fluorescence profile.

### Immunofluorescence

The immunocytochemistry using rabbit anti-human pan keratin ab was done by fixing the cells with 3.7% (w/v) formaldehyde in PBS for 10 minutes and rinsed in HBSS (3 × 5) minutes. To prevent unspecific binding, the cells were incubated with 1% BSA for 60 minutes. The slides were incubated overnight at 4 °C with antibody diluted in PBS containing 1% (w/v) BSA; rabbit anti-pan keratin (1/200). The cells were washed 3 × 5 minutes in HBSS before a 60 minutes incubation with Alexa Fluor 488® F(ab′)_2_ fragments of goat anti-rabbit IgG (Molecular Probes, Invitrogen Ltd, Carlsbad, USA). Following rinsing 3 × 5 minutes in HBSS, the cells were incubated with DAPI (1/1000) (Molecular Probes Invitrogen Ltd, Carlsbad, USA). For negative controls, the primary antibodies were omitted. The cells were imaged using delta vision fluorescent microscopy.

### Co-localization and internalization of bacteria

To assess bacterial internalization, bacteria were grown to reach the desired optical density (OD), then pelleted and washed with 0.9% NaCl. The bacterial pellet was resuspended in HBSS and stained with BactoView GFP (1 µL in 500 µL), followed by a 30-minute incubation in the dark. After staining, the bacteria were pelleted again, diluted, and prepared for exposure to cells. At each designated time point, the cells with and without bacteria were washed, stained with LTDR for 40 minutes, washed again, and imaged using DeltaVision fluorescence microscopy to evaluate bacterial internalization and co-localization.

### RNA extraction and reverse

Total RNA was extracted using RNeasy® plus mini kit 250 (QIAGEN 74136, Germany) following the manufacturer’s instruction. Genomic DNA was eliminated using gDNA eliminator. Subsequently, the RNA concentration and purity were measured using a Nanodrop spectrophotometer (ThermoFisher, Nanodrop 8000). After quantification, the RNA was stored at − 80 °C. The cDNA synthesis was performed using a High-Capacity RNA-to-cDNA kit (4388950) from Applied Biosystems (ThermoFisher), following the manufacturer’s instructions. The reactions were performed in a thermocycler (AB Applied Biosystems, GeneAmp PCR system) with a 20 μL reaction volume. Preheating was done at 65 °C for 5 minutes. The reaction then proceeded in the following order: incubation at 37 °C for 60 minutes and followed by 95 °C for 5 minutes and held at 4 °C. Finally, the cDNA was diluted three times by adding RNase-free water and then stored at − 20 °C until further use.

### Quantitative real time polymerase chain reaction (RT-qPCR)

RT-qPCR was performed using a Fast 7500 Real Time PCR system (Applied Biosystems, USA). The list of primers used is shown in Table 1. For each reaction, a total volume of 10 μL: 5 μL of SYBR green, 1.25 μL forward primer (5 μM), 1.25 μL reverse primer (5 μM), and 2.5 μL template cDNA was used. Each biological sample was run in duplicates per target gene. The thermocycler protocol was 50 °C for 2 minutes, 95 °C for 10 minutes, and 40 cycles of 95 °C for 15 s and 60 °C for 1 minute, and afterward, a melt curve was obtained to determine the changes in relative gene expression, actin was used as the housekeeping gene to normalize the expression of the target by the 2^–ΔΔCt^, where ΔCt is determined by subtracting the Ct value of actin from the Ct value of the target gene described by Livak and Schmittgen [27, 28]. Negative controls with no cDNA template were also included.

**Table 1:** Overview of primer sequences, accession numbers, primer efficiencies and relevant references

Gene	Forward primer sequence	Reverse primer sequence	Accession number	Efficiency (%)	Reference
*Actin*	CAGCCCTCCTTCCTCGGTAT	CGTCACACTTCATGATGGAGTTG	BT059604.1	103	This study
*tnfa1*	TGCTGGCAATGCAAAAGTAG	AGCCTGGCTGTAAACGAAGA	DQ787157.1	96.1	[22]
*il1b*	AGGACAAGGACCTGCTCAACT	CCGACTCCAACTCCAACACTA	AY617117	97.2	[23]
*cd68*	GAAATCCACGGCCAACTTAAC	CGGGTAGGAAAGATGGAAAGAA	NM_001165385.1	100	This study
*cd80/86*	TTGTCCCGCATCCATCTCACTGTAT	ATCTTGTCTGGCTTCACTATTTCTTG	DW580717.1	88,7	[24]
*mpo*	GTTTCCTGCCTGCTCAGTAT	GTTGCCAGGATACGGTTAGAG	BT072012.1	82	This study
*keratin13*	CGCATCTCTAGCTCCTCATTC	CCCTCCCATGCTGAAACTAA	XM_014131518.2	102	This study
*inos*	AGGTGCTGAATGTGTTGCAC	GTATTCTCCTGCCTGGGTGA	XM_014214976.2	78	[25]
*capg*	GAGACAGAGACAGGAAGAATGTG	GTGGAGAGAGAGAGAGAGGAAA	XM_014206775.2	100	This study
*mcsf*	CACCAGTAACCCTAACCACTTC	GACCTGCTTGTCCTGCATTA	NM_001171807.1	102	[26]
*mhcii*	GTGGAGCACATCAGCCTCACT	GACGCACCGATGGCTATCTTA	X70165	100	[24]

### Statistics

The statistical data analysis was performed by using GraphPad Prism 10 version 10.4.0 (GraphPad Software Inc), with a grouped analysis and Two-way ANOVA, with Tukey´s HSD test to compare the differences between the groups. The results were considered statistically significant when the p-value was less than *p* < 0.05.

## Results

Our previous studies showed that both salmon skin and corneal keratocytes engulfed plastic particles of varying size ranging from 400 nm and up to 10 µm [12, 29]. Furthermore, in preliminary trials we have found these cells to be capable of internalizing different inactivated fish pathogens [30]. As an extension, the current study aimed to study uptake of bacteria and their capacity to express immune-relevant genes during exposure to bacteria. Thus, this study focused on the pro-inflammatory and marker gene regulation during bacterial exposure.

### Flow cytometry and immunocytochemistry confirm a high proportion of keratocytes in scale-derived cells

**Fig. 2:**
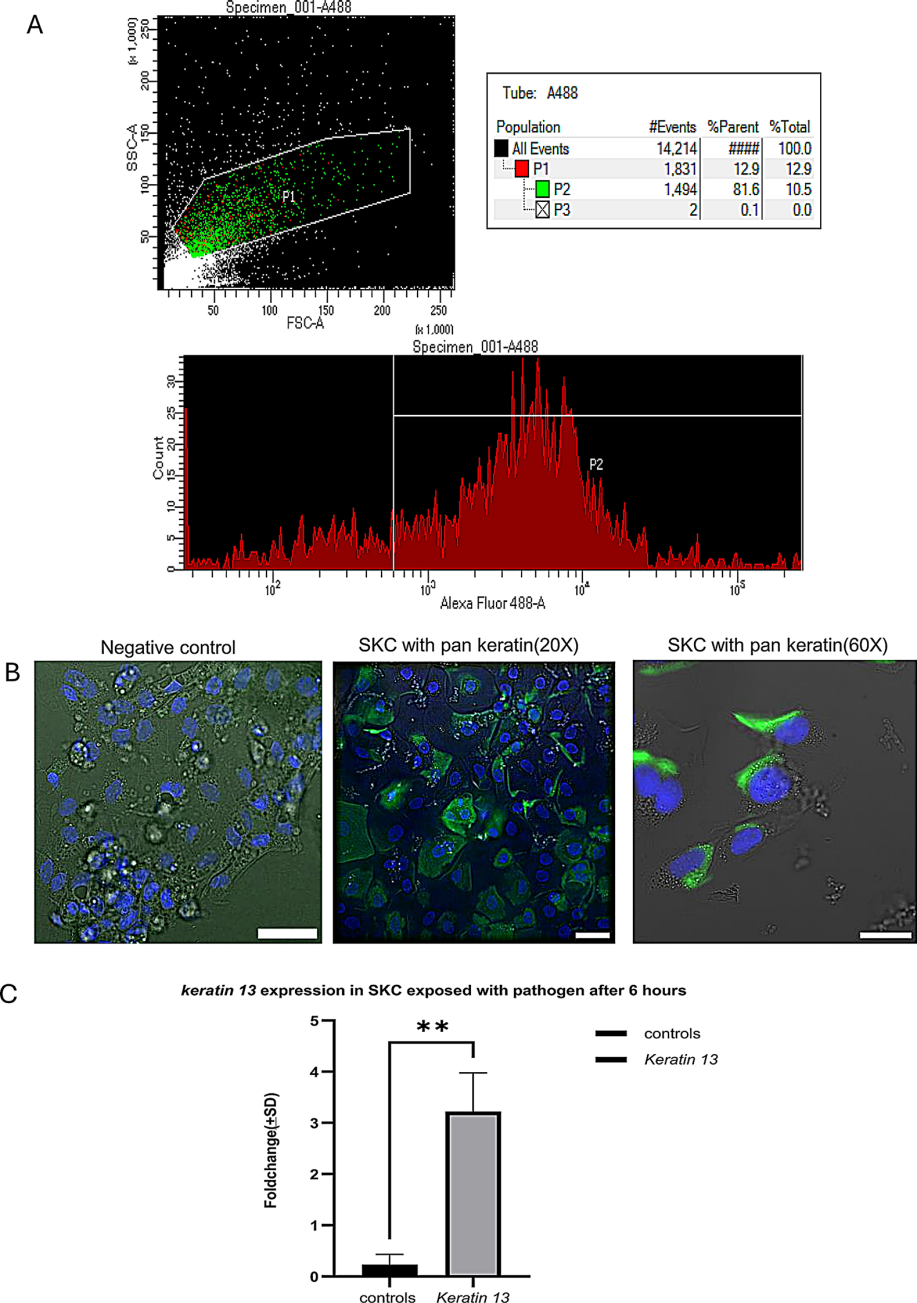
Flow cytometry showed that the scale cell population contained SKC majorly keratocyte cells. **A**) flow cytometer diagram showing most of the cell population stained for Alexa 488 (P2) compared to total cell population (P1). **B**) immunofluorescence staining of SKC with pan keratin antibody showing that most of the cells stained positively for pan keratin (20X/60X). Scalebar = 40 µm (20X)20 µm (60X). **C**) gene expression analysis showed at early timepoints most cells have keratocyte population and while cells exposed to pathogen showed a significant upregulation *kerati*n 13 gene compared to the control

The salmon skin epithelial layer comprises diverse cell populations, necessitating a proper analysis of the cell population derived from the scales. To address this, flow cytometry was used to analyze the cell population with special focus on pan keratin-labelled cells using a pan keratin antibody. Pan keratin has widely been used to label skin epithelial cells from higher vertebrates. The flow cytometry analysis showed that a high proportion of the cell population (~86,1%) gated with pan keratin antibody confirming that most of our cells were keratocytes (Fig. 2A). This result was confirmed by immunocytochemistry showing that these cells stained positively with pan keratin antibody (Fig. 2B). *Ker13* gene expression analysis also confirmed the existence of *keratin13* mRNA, which was significantly upregulated 6 hours after exposure to M. viscosa (Fig. 2C).

### Expression of pro-inflammatory cytokines during bacterial exposure

**Fig. 3:**
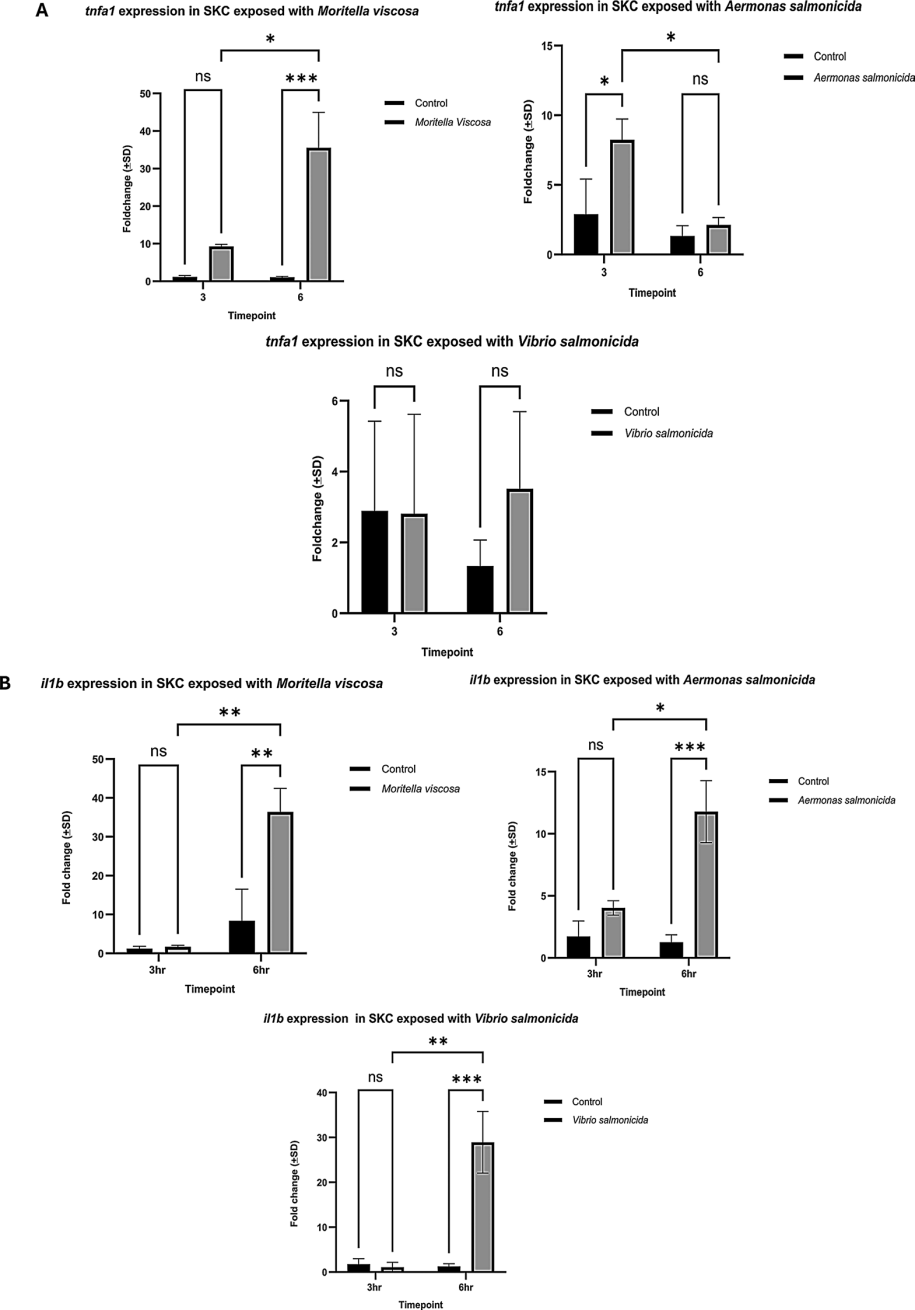
Expression of *tnfa1* and *il1b in SKC upon b*acterial infection. **A**) mRNA expression of *tnfa* in SKC exposed to different fish pathogens. **B**) mRNA expression of *il1b* in SKC exposed with different fish pathogens. Each bar represents mean relative expression data from triplicates ± SD. P-value <*0.05 considered as significant (grouped analysis and two-way ANOVA, with Tukey´s HSD), ns-nonsignificant

Inflammation is a critical process for recruiting immune cells during infection and tissue repair. To investigate the potential for inflammatory response elicited by cells, the expression of pro-inflammatory cytokines, including *tnfa1* and *il1b*, was analyzed following bacterial exposure. A significant upregulation of *tnfa1* following 3 hours exposure to *M. viscosa* and *A. salmonicida* was observed, with sustained expression at 6 hours during *M. viscosa* exposure (Fig. 3A). In addition, a significant upregulation of *il1b* was observed in cells exposed to three different pathogens at 6 hours (Fig. 3B). These results demonstrate that cells derived from scales actively express pro-inflammatory cytokines in response to exposure.

### Gene expression of markers is likely associated to macrophages

**Fig. 4:**
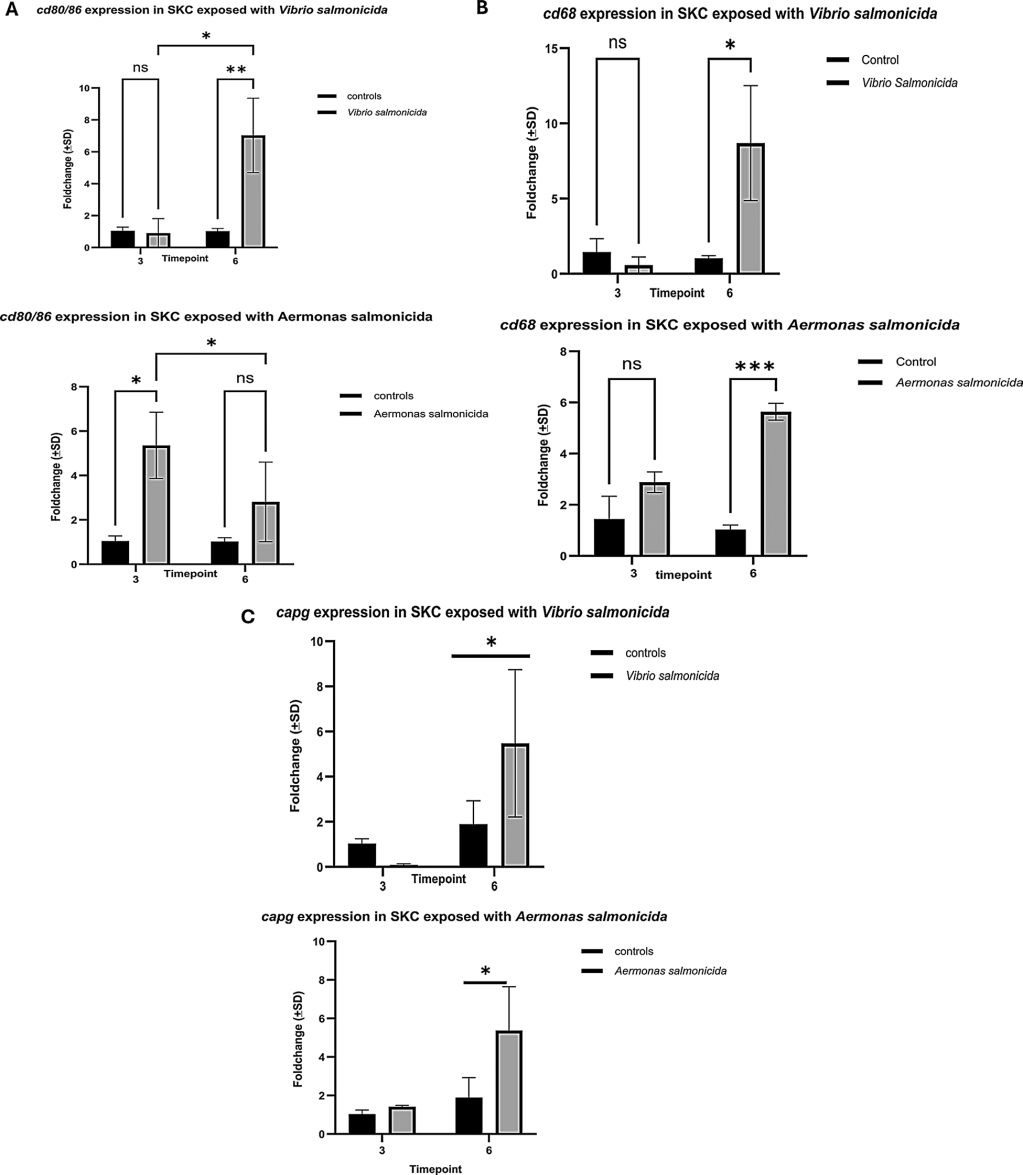
Represents the expression of cell surface markers upon bacterial infection. **A**) mRNA expression of *cd80/86* in cells exposed with different fish pathogens. **B**) mRNA expression of *cd68* in SKC exposed with different fish pathogens and **C**) mRNA expression of *capg* in the cell population. Each bar represents mean relative expression data from triplicates ± SD. *p* value <*0.05 considered as significant (grouped analysis and two-way ANOVA, with Tukey´s HSD), ns- nonsignificant

**Fig. 5:**
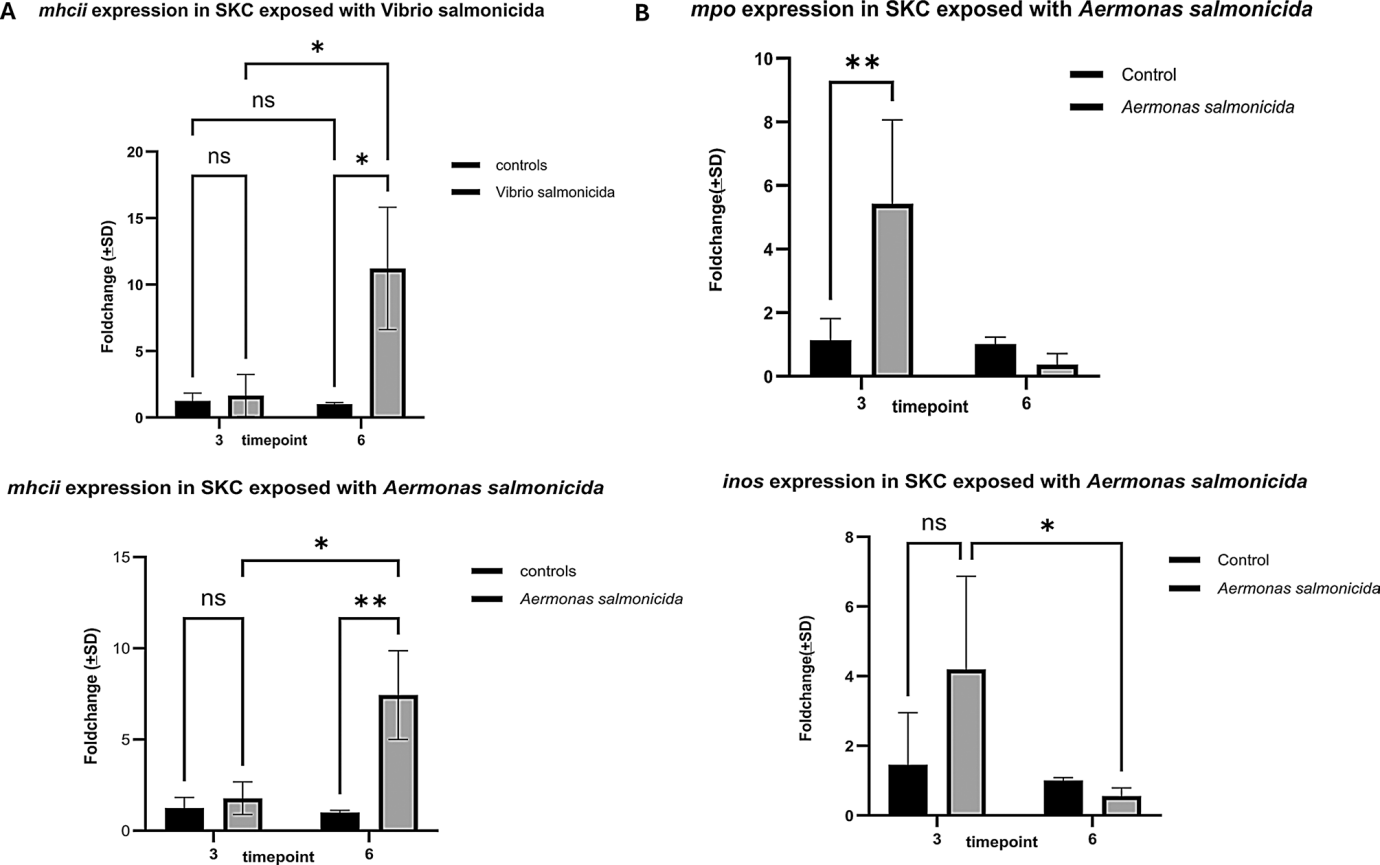
QPCR showing regulation of *mhcii, inos* and *mpo*. **A**) expression of *mhcii* in cells exposed to different fish pathogens. **B**) expression of *mpo* and *inos* exposed to A*. salmonicida.* each bar represents mean relative expression data from triplicates ± SD. *p* value <*0.05 considered as significant (grouped analysis and two-way ANOVA, with Tukey´s HSD), ns nonsignificant

As the scale-derived cell population may include a small proportion of other cells, we were interested in assessing expressions of macrophage marker genes such as *cd68*, *cd80/86, mcsf* and *capg in scale-derived cells.* To explore this, we analyzed their expression following bacterial exposure. *cd80/86* expression was significantly increased (Fig. 4A) and additionally a significant upregulation of *cd68* was observed at 6 hours *of* exposure to *V. salmonicida* (Fig. 4B). While the macrophage-specific genes such as *capg* (Fig. 4C) and *mcsf* (data not shown) also were upregulated, these changes were not statistically significant with exceptions - where *statistical* analysis *showed* significance between control cells and cells exposed to *A. salmonicida* and *V. salmonicida* at 6 hours (Fig. 4C). In addition, the scale cells expressed a significant level of *mhcii* mRNA (Fig. 5A) which is a specific marker for antigen presenting cells.

### Gene expression analysis of inflammatory markers in SKC

Inflammation induces expression of other pro-inflammatory markers. Thus, we wanted to look at RNA encoding *inos, mpo* and *mhcii -* how their expression is modulated following bacterial exposure. Inducible nitrogen synthetase (inos) is a macrophage product whereas myeloperoxidase (mpo) is secreted from neutrophils. Our gene expression data showed a significant upregulation of *inos* and *mpo* within 3 hours after exposure to different pathogens and a representative data following *V. salmonicida or A. salmonicida* exposure has shown in Fig. 5B. Interestingly, *mhcii* expression was significantly increased at 6 hours following exposure of *V. salmonicida* and *A. salmonicida* in Fig. 5A.

### Bacterial internalization in salmon keratocytes

**Fig. 6:**
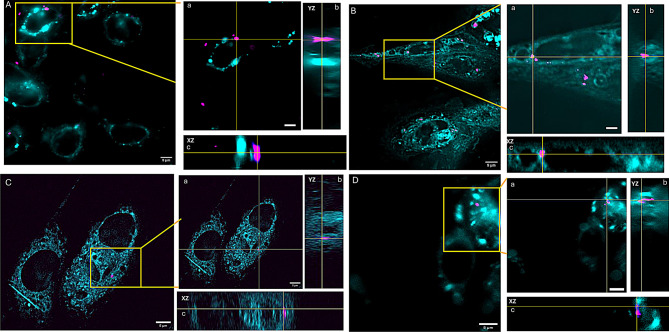
Colocalization of bacteria within lysosome in SKC and CKC. (**A**) *M. viscosa* bacteria (magenta) in SKC is shown clearly within the lysosome and touching a lysosomal marker (cyan) in the YZ plane (**A-b**). The colocalized region is shown as a single slice in XY (**A-a**) and its orthogonal planes: i.e. the view from the side of the 3D volume (**A-b/c**). Scale bars are (A left) 5 µm and (**A-a/b/c** right) 50 µm. (**B**) *M. viscosa* Bacteria(magenta) in CKC is shown clearly within the lysosome and touching a lysosomal marker (cyan) in the YZ plane (**B-b**). The colocalized region is shown as a single slice in XY (**B-a**) and its orthogonal planes: i.e. the view from the side of the 3D volume (**B-b/c**). Scale bars are (B left) 5 µm and (**B-a/b/c** right) 50 µm. **C**) *V. salmonicida* bacteria(magenta) in SKC is shown clearly within the lysosome and touching a lysosomal marker (cyan) in the YZ plane (**C-b**). The co-localized region is shown as a single slice in XY (**C-a**) and its orthogonal planes: i.e. the view from the side of the 3D volume (**C-b/c**). Scale bars are (C left) 5 µm and (**C-a/b/c** right) 50 µm. **D**) *V. salmonicida* Bacteria (magenta) in CKC is shown clearly within the lysosome and touching a lysosomal marker (cyan) in the YZ plane (**D-b**). The colocalized region is shown as a single slice in XY (**B-a**) and its orthogonal planes: i.e. the view from the side of the 3D volume (**D-b/c**). Scale bars are (D left) 5 µm and (**D-a/b/c** right) 50 µm

**Fig. 7:**
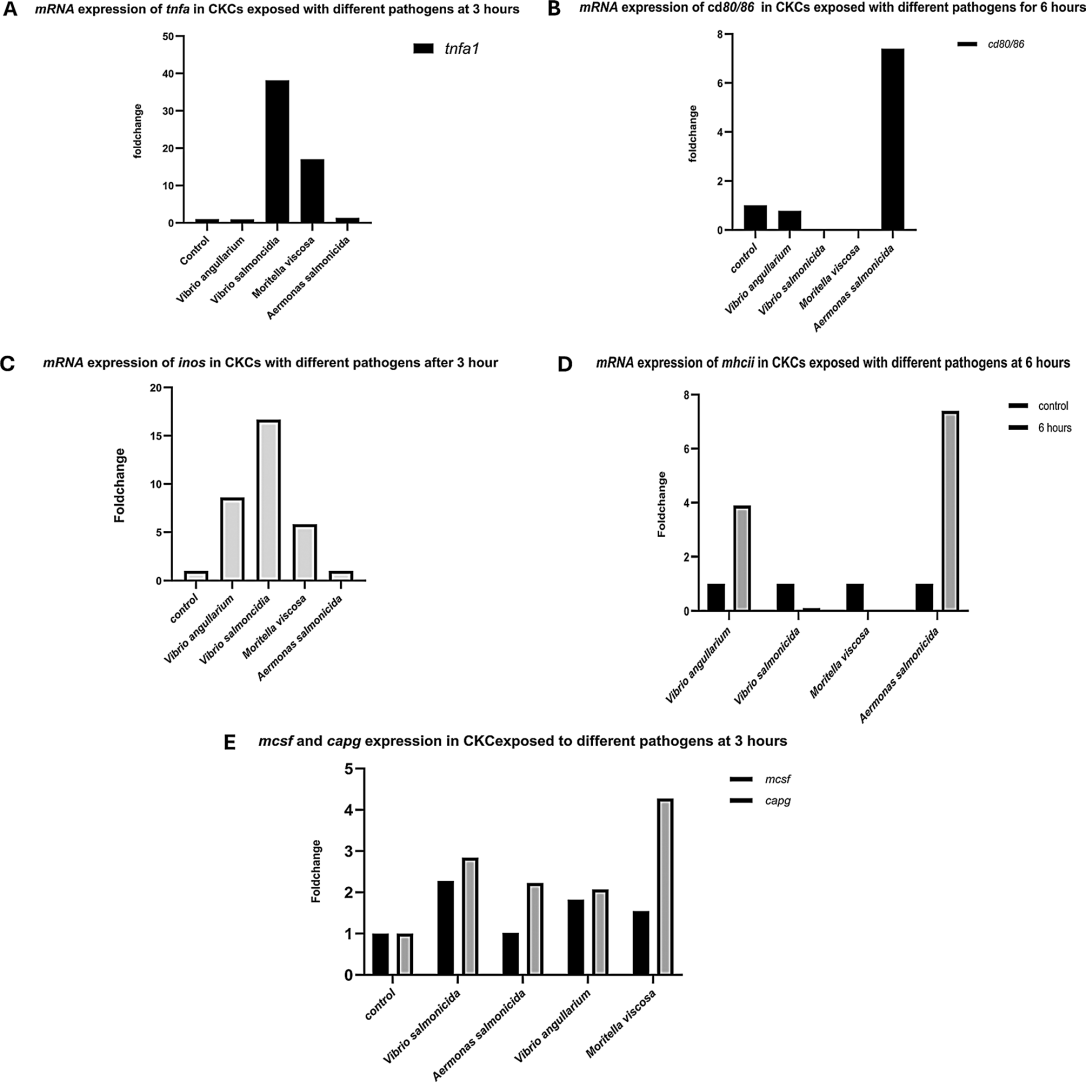
Immune gene expression of *tnfa1, mchii, inos* in CKCs after 3–6 hours of bacterial exposure. (**A**) expression of *tnfa1*; (**B**) expression of *cd80/86;* (**C**) expression of *inos;* (**D**) expression of *mhcii*; (**E**) expression of *mcsf and capg. Each bar represents relative expression data from a sample obtained from one individual eye*

Our previous studies [30] demonstrated that when SKC/CKC cells were exposed to dead pathogens, the pathogens were either internalized or associated with the cells. Gene expression analysis revealed that keratocyte cells upregulated several immune-related genes. To further investigate, we aimed to determine whether these cells could colocalize with lysosomes and exhibit phagocytic activity. Both SKC and CKC cells were exposed to *M. viscosa* labeled with BactoView GFP. After 6 hours, the cells were stained with a lysosome marker (LTDR) to assess colocalization. As shown in Fig. 6, colocalization of bacteria and lysosomes was observed in both SKC (Fig. 6A/C left) and CKC (Fig. 6B/D right) in their respective XY/ZY planes for SKC (6A-a/b/c) while for CKC the XY/ZY planes represented in (Fig. 6Ba/b/c).These results correlate with the upregulation of *mpo/inos* in both SKC (Fig. 5B) and CKC cells (Fig. 7C). Figure 6C/D shows the internalization of bacteria in both SKC and CKC exposed with *V. salmonicida* labelled with BactoView Live Green.

### Immune gene upregulation in corneal keratocytes upon infection

We speculated since the morphology of corneal epithelial cells (corneal keratocytes) was quite similar to skin epithelial cells (skin keratocytes), the expression of genes, as observed for skin keratocytes, was comparable. A similar pattern of immune gene expression was observed in corneal keratocytes upon bacterial infection (Fig. 7). Macrophage-specific markers including *cd80/86*, *tnfa1*, and the macrophage-associated gene *mcsf* were upregulated, within 3–6 hours of bacterial exposure. An upregulation was also observed at later timepoints (not shown). These results indicated that corneal keratocytes exhibit immune responses comparable to those of scale keratocytes, further underscoring their role in innate immunity. Since the numbers of corneal cells obtained from individual eyes/corneal layers were scarce, it was not possible to add multiple replicates in the qPCR experiments. Consequently, due to the lack of replicates, statistical significances with respect to fold-changes were not determined for the corneal qPCR data. Nevertheless, Fig. 6Aillustrates a higher fold change of *tnfa1* expression upon exposure to *V. Salmonicida* and *M. viscosa* compared to *V. anguillarum* and *A. salmonicida*. An upregulation of *inos* and *mpo*, in fold change of up to 20, was observed for corneal keratocytes - particularly in response to *V.* s*almonicida* exposure. The expression of macrophage-specific markers, *mcsf* and *capg* , was increased with fold changes 3–4 across all pathogens, with *capg* showing relatively higher expression compared to *mcsf*. Additionally, a notable fold change was observed in *cd80/86* expression upon bacterial exposure, especially when *A. salmonicida* was added to the cells.

## Discussion

The mucosal region of fish acts as a first line defense that initiates immune response upon any pathogen invasion [4, 7]. Furthermore, wound healing is an important process where the regeneration of fish skin takes place continuously and reconstitution of the epidermis is an important part of the healing process [6]. Keratocytes can cover fish skin wound surfaces with a new protective layer of cells within hours by rapid keratocyte migration from the surrounding wound margins [3, 13]. In addition, different cell types, including keratocytes, present in fish epidermis are shown to internalize bacteria and particles [31, 32]. Upon infection, local or acute inflammation is a general process initiated by resident cells to prevent pathogen entry and replication [33]. The fish skin epithelial layer is composed of mainly keratocytes and mucus producing cells [34]. However, interstitial leucocytes have also been observed [35]. The ratio of keratocytes to leucocytes was not examined in this study. However, in the current study by flow cytometry analysis revealed that the cell population predominantly consisted of keratocytes (approximately 86%), suggesting that the scale cell population also included a small proportion of leucocytes/other cells [34]. The analysis of other cell types within this cell population was challenging due to the lack of e.g., antibodies with cell-type specificity. Thus, the option was to use gene expression analysis. Having said that, Haugland et al. (2024) produced an antibody against salmon mhcii [24] - which will be used by us in future studies to assess mhcii-positive cells in skin and cornea.

Activation of cells by e.g., bacteria may induce *mpo* or *inos* activation [36] which implies that an active inflammation process that includes anti-microbial mechanisms. Myeloperoxidase is an enzyme produced by neutrophils [37], which is released to kill or inactivate pathogens by producing reactive oxygen species. Expression of *mpo* was evident in our cell population, which suggests that there may be presence of neutrophils. Nitric oxide (NO), that may catalyze mpo activity, is also a component of antimicrobial defense. Inducible nitric oxide synthetase can produce NO from L-arginine and is modulated during inflammation [38]. Further, studies have shown the presence of *inos* in teleost neutrophils [37]. Thus, the expression of both *mpo* and *inos* in cells from skin and cornea further supports their potential antimicrobial role in response to pathogen exposure. Additionally, our co-localization study demonstrated bacterial internalization within keratocytes, along with colocalization with lysosomes, which confirms their phagocytic function. Thus, the expression of genes in the current study may be from skin keratocytes together with other skin cells. We were not able to do a proper flow cytometry analysis of corneal cell population due to the low number of cells.

Here we have shown both salmon scale and corneal keratocytes have the potential to initiate an inflammation, by expression of pro-inflammatory cytokines, upon exposure to different bacteria, which in agreement with Kordon et al. how infection can trigger pro- inflammatory cytokines expression [39]. Studies have shown that pro-inflammatory cytokines, such as *il1b* and *tnfa*, are crucial in innate immune responses [40]. Following inflammation, particularly *il1b* and *tnfa* have been shown to be upregulated [21, 41]. However, fewer reports have focused on mucosal tissues and inflammatory responses mediated by these cytokines. Recently, through RNA sequencing it has been shown that innate immunity has been modulated in skin tissue after infection where there was expression of pro-inflammatory cytokines [42, 43]. The current study shows, for the first time, regulation of immune gene expression in keratocyte cells in vitro. From this, it appears that the scale and corneal keratocytes are capable of inducing inflammation by their expression of *tnfa* and *il1b* upon bacterial exposure. Both the skin and corneal keratocytes have been shown to be phagocytic against bacteria and plastic particles. Hence, we speculate that these cells may be non-professional phagocytic cells, or even non-professional antigen presenting cells. The latter is difficult to prove as there are no functional T cell assays available for A. salmon – to show how keratocytes can activate T cells. However here, we show that the scale-derived cells express *mhcii* mRNA, but whether the keratocytes express the mhcii protein is not yet known. If yes, we may categorize them as cells capable of antigen presentation. In addition, any presence of e.g., macrophages or dendritic cells (e.g., Langerhans cells) in skin would add the skin´s ability for proper antigen presentation and T cell response - dependent on the availability of T cells in the skin. To look specifically for macrophages, we employed a set of macrophage markers. Professional phagocytes such as macrophage and dendritic cells [44] induce upregulation of specific markers such as *mcsf*, *cd80/86* [24], *cd68* [45] upon exposure to bacterial antigens. Indeed, our study confirmed expressions of macrophage markers such as *capg*, *mcsf*. However, we were not able to discriminate against the expression between different cells in the population. We will in future studies try to sort the cells into different types followed by gene expression analysis. This will provide a more identification-based assessment whether the keratocytes also express some classical macrophage markers.

To date, there has been limited research focusing on corneal keratocytes in salmon. In contrast, extensive studies have been conducted in humans, highlighting their role in wound healing and inflammation during infection [46, 47]. One study in rainbow trout has shown a robust immune response upon infectious hematopoietic necrosis virus (IHNV) infection and bacterial infection in cornea [15, 16, 48]. Our study is the first to investigate how resident keratocytes respond to bacterial exposure. As mentioned, corneal keratocytes are able to ingest bacteria and express phagocytic markers showing its potential role antimicrobial defense upon infection. It is important to understand that the interaction of SKCs and CKCs with pathogens and cellular response during infection/internalization will pave a way for a better understanding of host pathogen interaction.

## Conclusion

In conclusion, this study sheds light on the critical role of salmon skin and corneal keratocytes (SKCs and CKCs) in immune responses, particularly their potential ability to initiate inflammation upon pathogen exposure. Our findings demonstrate that keratocytes not only express pro-inflammatory cytokines such as *il1b* and *tnfa* but also exhibit phagocytic activity, suggesting their potential as non-professional phagocytic and antigen-presenting cells. The expression of macrophage markers and immune-related genes further supports the hypothesis that keratocytes contribute to innate immune responses and their potential role in host-pathogen interactions.

## Electronic supplementary material

Below is the link to the electronic supplementary material.

Supplementary Material 1

## Data Availability

All data is freely available upon request from the corresponding author.
