# Supplementary material for: Immune gene expression in salmon keratocytes upon bacterial exposure
Source: BMC Mol Cell Biol. 2025 Sep 24;26:28. doi: 10.1186/s12860-025-00553-9 (PMC12462119; doi:10.1186/s12860-025-00553-9)
Supplement: Supplementary file 1 — Supplementary Material 1 [file 12860_2025_553_MOESM1_ESM.pdf]

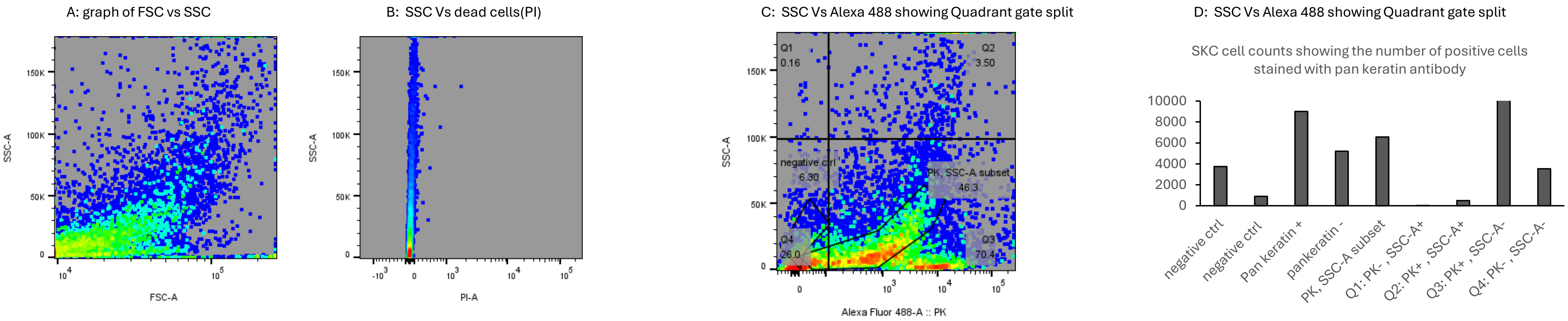

Supplementary Figure Legend:

Figure represents the gating strategy/gating tree of the cell population of SKC showing the pan keratin cell population.

A) FSC/SSC (represents the distribution of cells in the light scatter based on size and intracellular composition, respectively)

B) B)PI positive which represents the fraction of dead cells within the sample analyzed.

C) SSC/Alexa 488 quadrant gating split.

D) Cell count graph showing the number of positive stained cells Q1/2/3/4 subsets
